# Molecular and phylogenetic analysis of the porcine kobuvirus VP1 region using infected pigs from Sichuan Province, China

**DOI:** 10.1186/1743-422X-10-281

**Published:** 2013-09-11

**Authors:** Lei Chen, Ling Zhu, Yuan-cheng Zhou, Zhi-wen Xu, Wan-zhu Guo, Wen-yu Yang

**Affiliations:** 1 Animal Biotechnology Center, College of Veterinary Medicine of Sichuan Agricultural University, 46# Xinkang Road, Yucheng District, Ya’an 625014Sichuan province, P.R. China

**Keywords:** Porcine kobuvirus, VP1 protein, Diarrhea, Phylogenetic analysis, Co-infection, Recombination

## Abstract

**Background:**

Porcine kobuvirus (PKoV) is a member of the *Kobuvirus* genus within the *Picornaviridae* family. PKoV is distributed worldwide with high prevalence in clinically healthy pigs and those with diarrhea.

**Methods:**

Fecal and intestinal samples (*n* = 163) from pig farms in Sichuan Province, China were obtained to determine the presence of PKoV using reverse transcription polymerase chain reaction assays. Specific primers were used for the amplification of the gene encoding the PKoV VP1 protein sequence. Sequence and phylogenetic analyses were conducted to clarify evolutionary relationships with other PKoV strains.

**Results:**

Approximately 53% (87/163) of pigs tested positive for PKoV. PKoV was widespread in asymptomatic pigs and those with diarrhea. A high prevalence of PKoV was observed in pigs younger than 4 weeks and in pigs with diarrhea. Phylogenetic analysis of 36 PKoV VP1 protein sequences showed that Sichuan PKoV strains formed four distinct clusters. Two pigs with diarrhea were found to be co-infected with multiple PKoV strains. Sequence and phylogenetic analyses revealed diversity within the same host and between different hosts. Significant recombination breakpoints were observed between the CHN/SC/31-A1 and CHN/SC/31-A3 strains in the VP1 region, which were isolated from the same sample.

**Conclusion:**

PKoV was endemic in Sichuan Province regardless of whether pigs were healthy or suffering from diarrhea. Based on our statistical analyses, we suggest that PKoV was the likely causative agent of high-mortality diarrhea in China from 2010. For the first time, we provide evidence for the co-existence of multiple PKoV strains in one pig, and possible recombination events in the VP1 region. Our findings provide further insights into the molecular properties of PKoV, along with its epidemiology.

## Background

Members of the *Picornaviridae* are small, non-enveloped viruses with a linear single-stranded, positive-sense RNA genome. They are a highly diverse virus family, with a number of picornaviruses known to be important pathogens of humans and other animals [1-3]. The *Kobuvirus* genus is relatively new within the *Picornaviridae* family, and kobuviruses have been detected in humans, cattle, pigs, sheep, wild boars, bats, dogs, cats, goats and rodents [2-11]. The RNA genomes of kobuviruses range from 8.2–8.4 kb and contain one large open reading frame (ORF) encoding a single polyprotein. This polyprotein is cleaved into structural (VP0, VP3 and VP1) and non-structural (2A, 2B, 2C, 3A, 3B and 3D) proteins [12]. Currently, the *Kobuvirus* genus contains two officially recognized species, Aichi virus and bovine kobuvirus, with PKoV a candidate species. Aichi virus strain A846/88 was first isolated in Japan, in 1991, from the fecal sample of a patient suffering acute gastroenteritis [3]. Since then, Aichi virus has been detected in the fecal samples of humans from other countries in Asia, Europe, South America and Africa [13-16], and is thought to be associated with acute gastroenteritis. Bovine kobuvirus strain U-1 was detected in culture medium supplemented with calf serum possibly polluted with feces, and also in fecal samples from clinically healthy Japanese cattle in 2003 [2]. Bovine kobuvirus has also been detected in asymptomatic cattle from Hungary and the Netherlands, and in cattle from Thailand and Korea suffering diarrhea [17-19]. PKoV was detected in a Hungary farm in early 2007 and the complete genome (strain swine/S-1-HUN/2007/Hungary) characterized [4]. Since it was first identified, PKoV has been detected in China, Thailand, Spain, Japan, Korea, the United States, Brazil, the Netherlands, and recently in the Czech Republic [19-26]. The prevalence of PKoV-positive fecal samples ranged from 16.7–99% worldwide. On a single farm in Hungary, the prevalence among clinically healthy domestic pigs was 65% (39/60) and 53.3% (32/60) for 2007 and 2008, respectively [27]. In northern Spain, the rate of detection for PKoV was 48.7% in 2011 [22]. Epidemiological research of PKoV infection in Thailand revealed that 99% (97/98) of samples examined between 2001–2003 [21], and 97% (127/131) of samples from pigs suffering diarrhea between 2006–2008, were positive for the virus [28]. In healthy pigs from Japan, 45.4% (133/293) were positive for PKoV in 2010 [23]. Analysis of samples taken from eight Korean provinces showed that 84.5% (71/84) and 19.3% (16/83) of samples from pigs with or without diarrhea were positive for PKoV in 2010 [29]. Also in 2010, PKoV was reported in 45.5% (15/33) and 32.6% (28/86) of non-diarrheal and diarrheal samples, respectively from three Korean provinces [24]. In Brazil and the Netherlands, prevalence was reportedly 53% (61/115) and 16.7% (3/18), respectively [19]. On USA farms, PKoV infection rates were 21.9% (25/114) in pigs with diarrhea and 21.7% (10/46) for apparently healthy pigs [25]. For healthy pigs from the Czech Republic, the overall prevalence was 87.2% (171/196) [26]. In China, PKoV was detected in 38.8% (45/116) and 30.12% (97/322) of samples from Shanghai and Lulong County, respectively [30,31].

PKoV is an emerging virus prevalent worldwide in healthy pigs, and in pigs with diarrhea. The pathogenicity of PKoV remains unclear as culturing of the virus *in vitro* has not been successful. A recent study from Korea suggested that PKoV might be the etiological agent of gastroenteritis in pigs [29]. Most molecular epidemiological investigations of PKoV have focused on the 3D region, while information regarding the molecular properties of the gene encoding the VP1 protein is relatively limited. The VP1 region is the most exposed and immunodominant portion of the capsid protein, and is the most variable structural protein among all kobuviruses [2,32]. It plays an important role in the molecular epidemiology and genetic evolution of kobuviruses. Yu *et* al. identified 11 PKoV VP1 sequences in piglets, from Lulong County China, younger than 15 days; similarities among these sequences ranged from 86.7–100% [30]. Okitsu *et* al. described the genetic diversity of PKoV VP1 in Japan and Thailand [28]. Recently VP1 protein sequences were used to investigate the relatedness among PKoV strains by Shi *et* al.; their findings indicated that known PKoV strains form four distinct lineages [33].

Further epidemiological and molecular research on PKoV strains are required to assist researchers and health officials to identify its epidemic characteristics, distribution, evolutionary features and genome sequences. The aim of our study was to determine the prevalence of PKoV in Sichuan Province China, and to analyze the phylogenetic and genetic relationships of the VP1 region between Sichuan PKoV and reference kobuvirus strains. Statistical analysis was also conducted to determine any association between PKoV infection and the age of pigs, or symptoms of diarrhea.

## Results

### Prevalence of PKoV in pigs of Sichuan Province

Using primers targeting the 3D region, 53.4% (87/163) pigs were positive for PKoV. The proportion of asymptomatic pigs and those with diarrhea that were PKoV-positive were 29.4% (15/51) and 64.3% (72/112), respectively. For the samples from pigs with diarrhea, 76.5% (52/68) were from suckling pigs, 46.7% (7/15) were from weaned pigs, 30% (3/10) were from growing/finishing pigs, and 52.6% (10/19) were from sows. Among the PKoV-positive clinically healthy pigs, 40% (10/25), 25% (2/8), 14.3% (1/7), and 18.2% (2/11) were suckling pigs, weaned pigs, growing/finishing pigs, and sows, respectively (Table 1). PKoV was detected in 17 of the 18 cities sampled. None of the fecal samples from apparently healthy Tibetan pigs were positive for PKoV. Statistical analysis showed that porcine kobuvirus infection strongly correlated with suckling pigs, which were younger than 4 weeks old (*χ*^2^ = 10.941, *p* = 9.4 × 10^-4^). PKoV infection was significantly associated with diarrhea in pigs (*χ*^2^ = 17.126, *p* = 3.5 × 10^-5^) using Pearson’s chi-square test.

**Table 1:** Prevalence of Porcine kobuvirus in pigs from Sichuan Province

**Pig age**	**Diarrheic pigs**	**Non-diarrheic pigs**	**Total**
Suckling pigs	52/68^a^(76.5%)	10/25(40.0%)	62/93(66.7%)
(<4 weeks, n = 93)			
Weaned pigs	7/15(46.7%)	2/8(25%)	9/23(39.1%)
(< 7 weeks, n = 23)			
Growing/finisher pigs	3/10(30.0%)	1/7(14.3%)	4/17(23.5%)
(<6 months, n = 17)			
Sows	10/19(52.6%)	2/11(18.2%)	12/30(40.0%)
(>1 year, n = 30)			
Total(n = 163)	72/112(64.3%)	15/51(29.4%)	87/163(53.4%)

^a^ Number of porcine kobuvirus RNA positive samples/tested samples.

### Sequence and phylogenetic analysis

Among the 87 PKoV-positive samples, 32 were randomly selected for amplification of the gene encoding the VP1 protein. We obtained 30 partial PKoV VP1 sequences using a nested PCR technique. Nucleotide and deduced amino acid similarities among these 30 partial sequences ranged from 82–100% and 86.9–100%, respectively. We detected multiple strains of PKoV in two samples (pigs 31 and 32) following sequence examination of first-round PCR amplicons. We detected three PKoV strains within pig 31, based on the presence of three different 1190 bp fragments, designated A1, A2, and A3. The nucleotide and deduced amino acid identities ranged from 97.5–98.8% and 98.2–98.7%, respectively. The 1190 bp fragment covered the full VP1 region (nt 182–943), a portion of VP3 (nt 1–181), and a part of the 2A region (nt 944–1190). For pig 32, sequencing results revealed two different PKoV strains, designated B1 and B2. Sequence analysis revealed that B1 and B2 were significantly diverse at the genetic level with 84.5% and 90.7% identity at the nucleotide and amino acid level, respectively. We submitted these 35 PKoV VP1 gene sequences that were analyzed to GenBank (Accession numbers: KF157917–KF157951).

**Figure 1:**
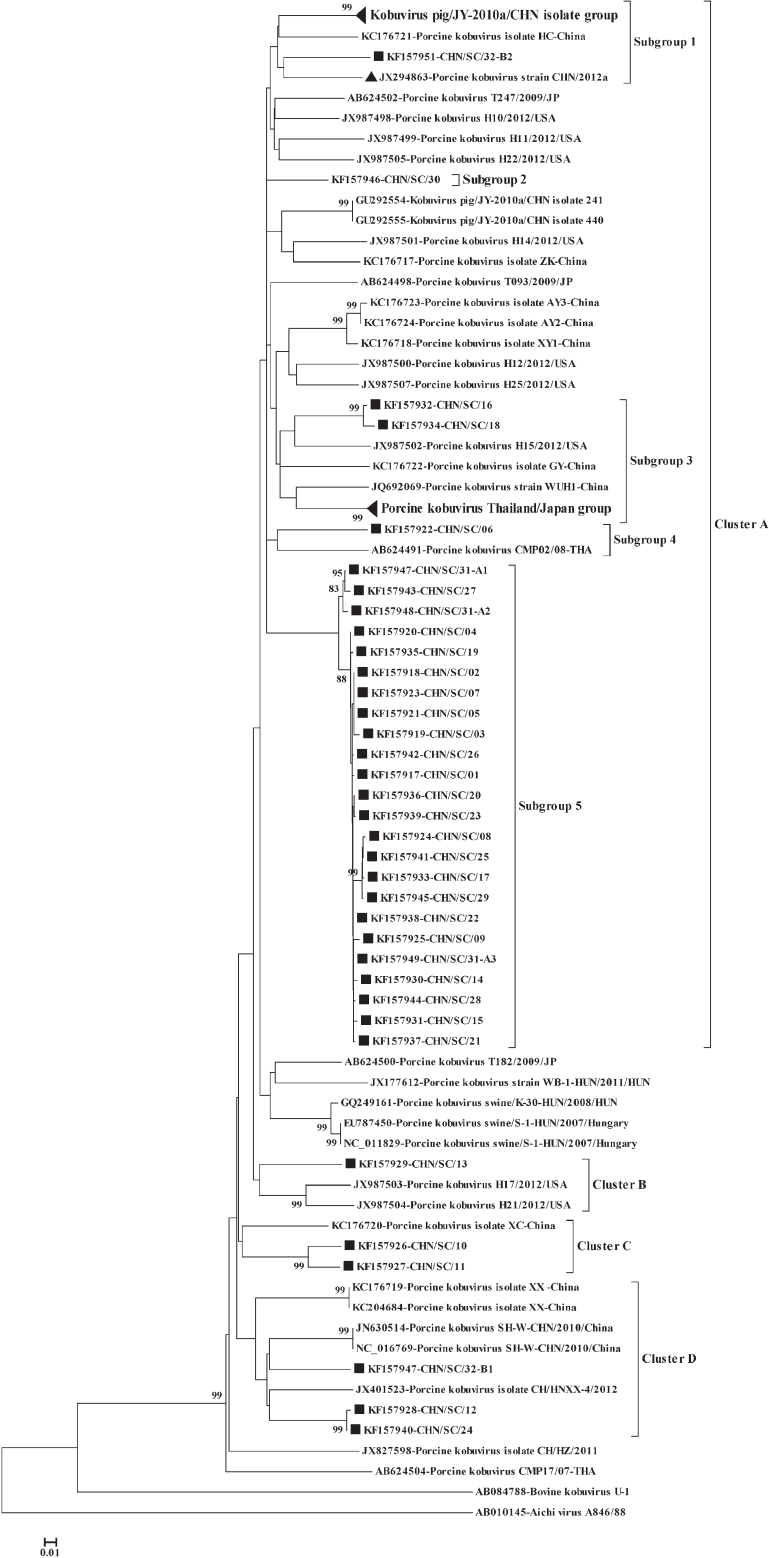
**Phylogenetic analysis of PKoV VP1 protein sequences detected in Sichuan province.** The phelogenetic tree was constructed using the neighbor-joining method, Kimura 2-parameter model by MEGA version 5.0. Bootstrap values (based on 1,000 replicates) >75% are shown. Filled quadrate (■) indicates PKoV VP1 protein sequences obtained in this study. Filled triangle (▲) represents a Sichuan strain PKoV VP1 protein sequence has been reported in our previous study. Kobuvirus pig/JY-2010a/CHN isolate group contained 10 sequences from China forming a tight cluster. The Genbank accession numbers are: GU292548-GU292553, GU292556-GU292559; PKoV Thailand/Japan isolate group including 10 sequences clustered in a monophyletic branch. Their accession numbers in Genbak are as follows: AB624490, AB624492-AB624497, AB624499, AB624501, AB624503.

A phylogenetic tree was generated using the 35 sequences we determined. In several genera of the *Picornaviridae*, similarities at the nucleotide and amino acid level for VP1 are used as criteria for taxonomic classification. The phylogenetic tree indicated that the 35 sequences we submitted to GenBank were indeed PKoV strains (Figure 1). A neighbor-joining tree based on the VP1 gene indicated that the 35 Sichuan PKoV strains could be divided into four large clusters (Clusters A, B, C, and D) (Figure 1). JX294863 (CHN/2012a) was a PKoV VP1 sequence we submitted to GenBank in 2012, which was also isolated from Sichuan Province. Most Sichuan strains (30/36) were located in Cluster A, along with PKoV reference strains from China, the United States, Japan, and Thailand. Cluster A could be divided further into five subgroups: 11 VP1 sequences from China along with those for CHN/2012a and CHN/SC/32-B2 formed subgroup 1. Nucleotide identities within subgroup 1 were 84.7–100%, while amino acid similarities were 92.2–100%. Subgroup 2 comprised CHN/SC/30 only, while subgroup 3 contained CHN/SC/16, CHN/SC/18, two Chinese PKoV VP1 sequences, H15/2012/USA and PKoVs from Thailand and Japan. CHN/SC/06 clustered into subgroup 4 and was closely related to CMP02/08-THA, and subgroup 5 was made up of 24 Sichuan strains. Within subgroup 5, these VP1 sequences were 97–100% similar at the nucleotide level, and 95.4–100% similar at the amino acid level. CHN/SC/13 was highly similar to PKoV strains previously discovered in the United States and in Cluster B, sharing 85.6% and 87.3% nucleotide identity with H21/2012/USA and H17/2012/USA, respectively and greater than 95% identity with the deduced amino acid sequences. CHN/SC/11 and CHN/SC/10 were closely grouped together in Cluster C, along with the PKoV isolate XC from China. CHN/SC/11 and CHN/SC/10 sequences were compared with that for the XC isolate and found to be 86.2% and 84.2% similar, respectively, at the nucleotide level; amino acid identities were 94.5% and 92.2% similar, respectively. For Cluster D, CHN/SC/12, CHN/SC/24 and CHN/SC/32-B1 clustered with the PKoV reference strains from China (isolate XX, SH-W/CHN/2010/China, and CH/HNXX-4/2012).

CHN/SC/31-A1 (KF157947),CHN/SC/31-A2 (KF157948), and CHN/SC/31-A3(KF157949), which were isolated from pig 31, formed a single cluster (Figure 1), whereas, CHN/SC/32-B1 (KF157950) and CHN/SC/32-B2 (KF157951) were in two distinct groups. The five 1190-bp polyprotein sequences were compared with the corresponding region in eight complete PKoV sequences. Nucleotide and amino acid similarities ranged from 82.1–88% and 88.9–95.7%, respectively (Table 2).

**Table 2:** Nucleotide and amino acid identities of the polyprotein gene sequences

**Strain**	**Nucleotide identity (%)**												
	**CHN/SC/31**	**CHN/SC/32**	**CHN/SC/33**	**CHN/SC/34**	**CHN/SC/35**	**S-1-HUN**	**Y-1-CHI**	**SH-W-CHN**	**WUH1**	**WB-1-HUN**	**HNXX-4**	**CH/HZ/2011**	**XX**
CHN/SC/31	—	98.3	97.5	85.1	86.7	87.8	87.8	84.4	86.1	84.7	85.2	82.8	84.0
CHN/SC/32	98.7	—	98.8	85.3	86.8	87.4	87.3	84.4	86.3	84.7	85.1	82.1	84.0
CHN/SC/33	98.2	98.7	—	85.7	87.3	87.2	87.6	84.4	86.5	84.8	85.3	82.5	84.4
CHN/SC/34	91.9	91.7	92.4	—	84.5	84.5	85.4	87.9	84.5	83.6	88.0	83.4	86.9
CHN/SC/35	95.7	95.2	95.5	90.7	—	87.2	87.9	84.5	86.3	85.1	83.4	82.2	83.7
S-1-HUN	95.7	95.7	95.5	91.4	95.5	—	86.0	84.3	86.9	86.6	83.4	84.4	84.8
Y-1-CHI	94.2	94.2	94.2	92.2	95.7	94.9	—	86.2	87.3	84.4	84.8	83.3	84.4
SH-W-CHN	89.9	89.6	89.9	93.9	88.9	88.6	89.6	—	84.5	82.8	86.5	84.0	86.4
WUH1	95.7	95.5	95.2	91.4	93.7	96.0	93.9	89.1	—	85.1	84.2	83.4	85.5
WB-1-HUN	92.4	92.2	91.9	89.9	90.7	93.4	91.9	87.1	93.7	—	83.0	81.1	84.5
HNXX-4	91.2	90.9	91.4	96.5	89.9	90.9	91.4	92.7	90.7	90.4	—	83.5	87.7
CH/HZ/2011	90.9	90.7	91.2	92.7	90.4	90.7	91.9	90.9	90.9	89.1	91.7	—	83.9
XX	90.4	90.2	90.4	94.4	88.9	90.2	89.9	91.9	91.7	90.2	93.9	90.9	—
Amino identity (%)													

The reference porcine kobuvirus used in the comparison and their accession numbers are as follows: S-1-HUN (EU787450), Y-1-CHI (GU292559), SH-W-CHN (JN630514), WUH1 (JQ692069), WB-1-HUN (JX177612), HNXX-4 (JX401523), CH/HZ/2011 (JX827598), XX (KC204684).

### Recombination analysis

**Figure 2:**
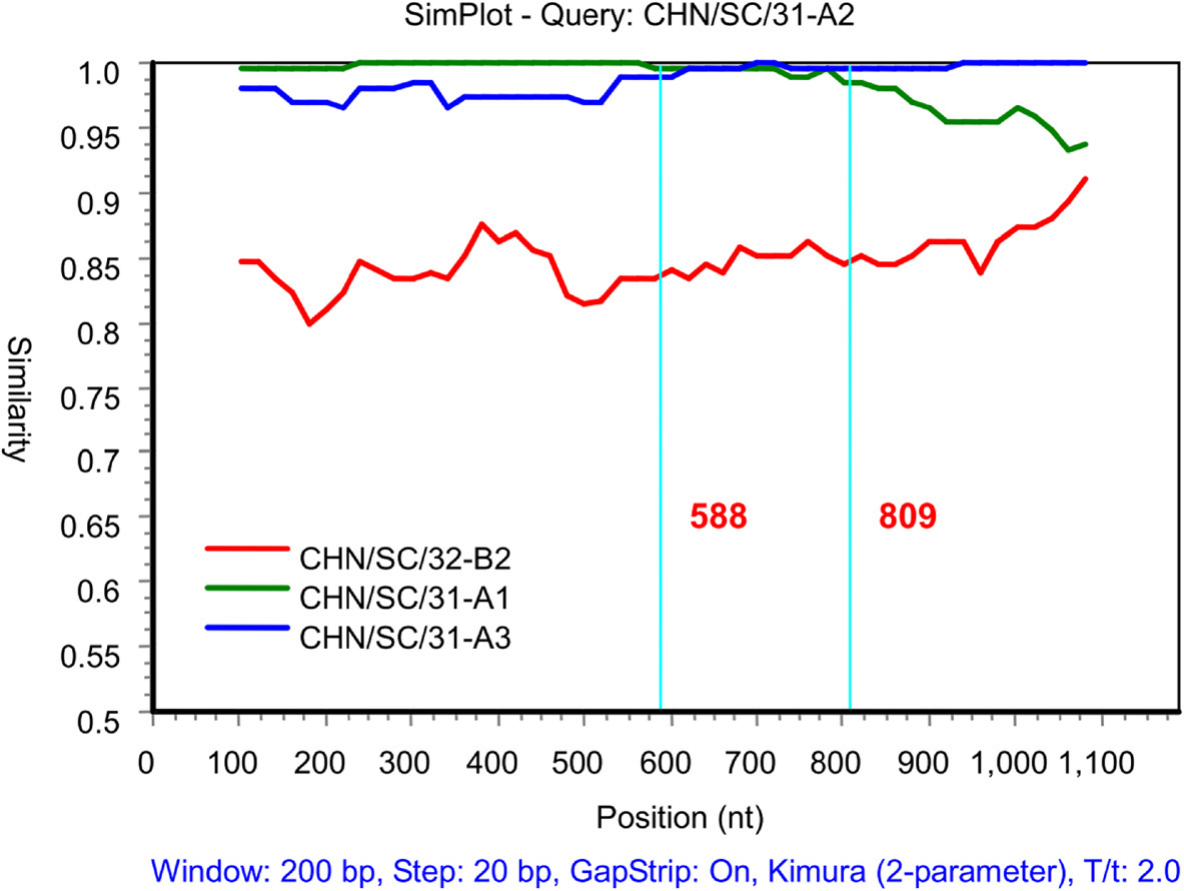
**Nucleotide similarity comparison between the putative parental strains and the possible recombination strain.** CHN/SC/31-A1 and CHN/SC/31-A3 were the putative parental strains, and CHN/SC/31-A2 was the query sequence, CHN/SC/32-B2 was an outgroup. The x-axis indicates nucleotide positions, and the y-axis shows similarity.

**Figure 3:**
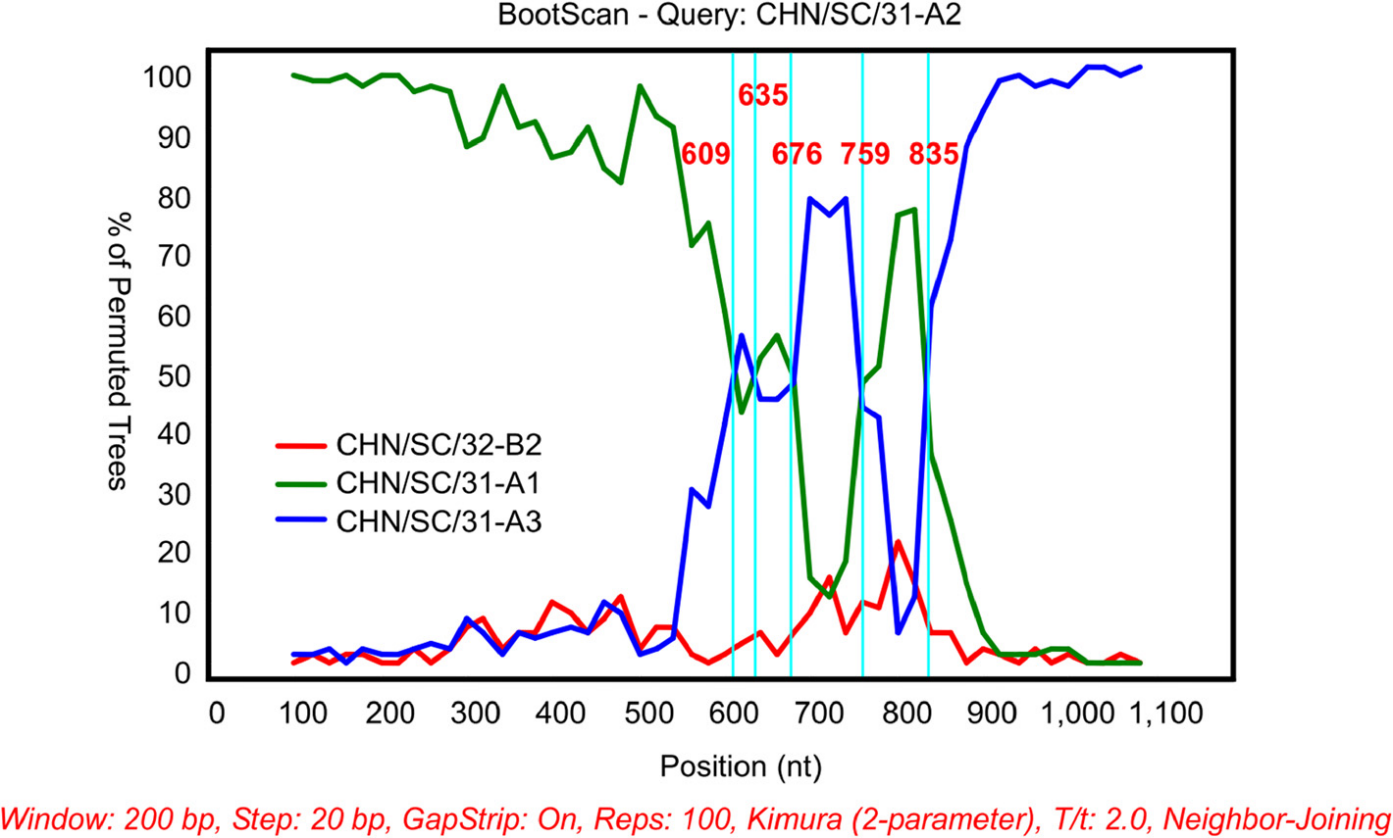
**Bootscan analysis of the putative recombinant sequence.** The x-axis indicates nucleotide positions. The y-axis denotes bootstrap values that support the clustering of CHN/SC/31-A2 with the parental strains.

Significant recombination breakpoints were detected for different polyprotein gene sequences isolated from the same host using sequence alignment and software analyses. We observed recombination events in the three polyprotein sequences from pig 31 using the Recombination Detection Program (RDP) and its automated suite of algorithms, including GENECONV, MaxChi, Chimaera, SiScan and BootScan [34-38]. At least four sequences were required to run the Simplot program [39]. On the basis of the RDP analysis results, both CHN/SC/31-A1 and CHN/SC/31-A3 were identified as the putative parental strains, CHN/SC/31-A2 was a possible recombinant strain, and CHN/SC/32-B2 was regarded as an outgroup. The Simplot analysis results confirmed possible recombination events among the different polyprotein sequences from pig 31. The similarity plot (Figure 2) displays consecutive nucleotide identities between the queried and parental strains. The recombined CHN/SC/31-A2 strain exhibited greater similarity to CHN/SC/31-A1 upstream of nt 588, but shared higher sequence similarity with CHN/SC/31-A3 downstream of nt 809 (Figure 2). Between nucleotides 588 and 809, we observed crossovers sites for the two putative parental sequences. Essentially, the parental sequences shared equal identity to the query strain, and we postulated that these were the sites of recombination. BootScan analysis showed potential recombination breakpoints between nt 609–835 (Figure 3). Before the first breakpoint, near nt 609, high bootstrap values for clustering of CHN/SC/31-A1 with CHN/SC/31-A2 were observed. In contrast, after the last breakpoint, near nt 835, CHN/SC/31-A2 clustered more closely with CHN/SC/31-A3. Some changes that affected clustering between nt 609–835 were observed, with nt 609–635 and 676–759 of CHN/SC/31-A2 more closely related to CHN/SC/31-A3, while nt 635–676 and 759–835 of CHN/SC/31-A2 were more likely to cluster with CHN/SC/31-A1. These results were in accordance with the results from the quick trees generated by Simplot.

## Discussion

Our findings indicate that PKoV-infected pigs are not restricted geographically but distributed worldwide regardless of clinical conditions. In the present study, we determined PKoV infection status and prevalence in healthy pigs and in those suffering diarrhea from Sichuan Province, China. PKoV was detected in 17/18 sampled cities. Around 53% (87/163) of samples contained PKoV, with 64.3% (72/112) of diarrhea samples and 29.4% (15/51) of normal samples containing the virus. These results indicate general circulation and endemic infection of PKoV in Sichuan domestic pigs. We also confirmed that PKoV is common in apparently healthy pigs, which agrees with the findings presented by researchers in other countries. The infection rate in healthy pigs (29.4%) was similar to that seen in Lulong County (30.12%), slightly higher than that for Shanghai (22.4%) [31], and the United States (21.7%) [25], and much lower than that for Hungary (65%) [27]. In pigs with diarrhea, prevalence was 64.3% and in accordance with that previously reported in Shanghai (61.2%) [31], and much lower than that observed in Thailand (97–99%) [21,28], Brazil (78.4%) [19] and Korea (84.5%) [29]. These differences in prevalence can be largely attributed to sampling time, sampling range, fecal consistency, and age of evaluated pigs.

Statistical analysis of PKoV incidence suggests that PKoV infection correlates with diarrhea (*χ*^2^ = 17.126, *p* = 3.5 × 10^-5^). Similar results have been reported in Shanghai (*p* = 0.000), Brazil (*p* = 0.0002) and Korea (*p* = 3.2 × 10^-17^) using Pearson’s chi-square test [19,29,31]. However, it was not possible to conclusively show that PKoV was the etiological agent of diarrhea. The existence of other pathogens that can cause diarrhea could not be ruled out. In three diarrhea samples from Korea that tested positive for PKoV, other enteric pathogens were not detected [29]. There was a high prevalence of virus in pigs with diarrhea from Thailand (97–99%) [21,28] and Korea (84.5%) [29]. These observations imply that PKoV might have some association with diarrhea in pigs. The lack of a cell culture system to propagate PKoV *in vitro* limits further study regarding the biology and pathogenicity of this emerging virus.

Among the four tested age groups, piglets under the age of 4 weeks were more likely to be infected with PKoV (*χ*^2^ = 10.941, *p* = 9.4 × 10^-4^), which is similar to what has been reported in Hungary, Shanghai, the United States and Brazil. These reports indicate that young piglets are highly susceptible to PKoV infection [19,25,27,31]. A study conducted by Barry *et* al. indicated that this might be possibly be due to an inefficient immune response or other intrinsic age-related factors [19].

Prevalence of PKoV in suckling, weaned, and growing/finisher pigs decreased as host age increased. Similar patterns have been observed in swine herds from Hungary, Korea, the United States, Brazil, and Japan [23-25,27,29]. In the Czech Republic, higher PKoV prevalence in samples from post-weaning pigs and nursing piglets were seen compared with those obtained from an abattoir [26]. Therefore, PKoV infection might have an association with host age. Further epidemiological studies from other geographical areas will be required to clarify this.

The overall frequency (40.0%) of PKoV in sows is much lower than that reported by Dufkova *et* al. (90.9%), but much higher than that reported by Barry *et* al. (11.8%) [19,26]. According to these researchers, unproductive infection and passive shedding of the virus might result in the low prevalence among sows living in the same breeding environment. However, these conditions can lead to a high prevalence in piglets, as infected sows might act as a reservoir of PKoV and cause continuous infection of piglets.

Kobuvirus was not detected in any of the Tibetan pigs we sampled. It is possible that PKoV infection is not as frequent in Tibetan pigs compared with that observed in domestic pigs. However, as only four pigs were examined it is more likely that this number is not representative of the actual infection status.

The VP1 sequences identified in Sichuan Province formed four large clusters, suggesting multiple PKoV strains are circulating in Sichuan Province. Previous studies have already revealed the existence of multiple PKoV lineages in China and Korea [21,24,31]. Our phylogenetic analyses confirmed high levels of genetic diversity for the VP1 gene, which has been previously reported [28,32,40].

We found that two pigs (pigs 31 and 32) were co-infected with multiple PKoV strains. Three different PKoV strains were found in pig 31, and two different PKoV strains in pig 32. This is the first report, with supporting evidence, of multiple strains of PKoV co-infecting a single pig. On a Brazilian farm, different PKoV strains were suspected to exist in a serum sample from an individual pig, but only one strain was detected. Identification of the strain was based on a gene that was more conserved than that for VP1. The authors of this report suggested it was likely that there were indeed two different strains and those they were not only a result of constant changes in the RNA genome of kobuviruses [19]. CHN/SC/31-A1, CHN/SC/31-A2 and CHN/SC/31-A3 (obtained from pig 31) formed a cluster. CHN/SC/32-B1 and CHN/SC/32-B2 belonged to different branches, sharing 84.5% nucleotide sequence identity. This would suggest genetic diversity of PKoV within the same host and between different hosts. This is also the first report describing marked sequence diversity of PKoV within the same host.

Recombination plays an important role in the evolution of virus genomes. It is a major driving force for the generation of new genotypes or strains of virus. Significant recombination breakpoints were observed in the polyprotein gene sequences for pig 31. Two parent strains simultaneously infecting one host is a prerequisite for recombination. Co-infection of multiple PKoV strains in the same pig may potentially facilitates the occurrence of recombination events. Possible recombination events were analyzed for CHN/SC/31-A2; these might have been generated from recombination between CHN/SC/31-A1 and CHN/SC/31-A3 in the VP1 region. Recombination events have contributed to the genetic diversity within hosts that we observed. Multiple PKoV strains in the same pig could have arisen from recombination events [41].

Wang *et* al. used Simplot for genetic analysis of PKoV strains; however, no significant recombination events were identified in SH-W-CHN, the strain they investigated. Certain possible recombination signals were identified in a small region (nt 8083–8210) at the 3′ end of the viral genome [31]. BootScan results and phylogenetic analysis of five complete Aichi virus sequences revealed a mosaic genome sequence of Aichi virus [42]. Phylogenetic analysis of the VP1 region and 3D region of strain H023/2009/JP suggest it may be a natural recombinant from porcine and bovine kobuviruses [23,28]. Recombination in kobuviruses is likely to be a usual phenomenon, just as it is in other members of the Picornavirus genus [43].

PKoV infection is widely distributed in healthy pigs and asymptomatic pigs, providing favorable conditions for recombination [31]. The pathogenic and zoonotic potential of PKoV remains unclear. The closely related Aichi virus is the causative agent of human gastroenteritis, and bovine kobuvirus is associated with diarrhea in cattle [3,12,17,44-46]. It would appear that the pathogenesis of PKoV is similar to that for other picornaviruses, and we believe that it may have a major role in causing enteric diseases in swine.

Since the end of 2010, massive outbreaks of diarrhea have occurred in suckling piglets in China; however, the etiological agent has yet to be determined. Affected pigs exhibited signs of watery diarrhea, dehydration, and vomiting with morbidity ranging 80–100% and mortality between 50–90%. Of the suckling pigs we tested in this study, 76.5% (52/68) were suffering from diarrhea and positive for PKoV. A high frequency of PKoV in piglets with gastroenteritis has been observed in other countries. Therefore, we propose that PKoV is the likely etiological agent of these outbreaks of severe diarrhea in China that began in 2010.

Recombination in kobuviruses creates changes in virus genomes, which probably generates new virus variants. Cao *et* al. sequenced and analyzed the complete genome of a PKoV variant with a 30-amino acid deletion in the 2B-coding region and a threonine amino acid insertion in its VP1 region. This variant was isolated from the 2010 outbreak in China [47]. It is possible that PKoV variants generated through recombination or other evolutionary forces are related to the large-scale outbreak of severe diarrhea in suckling piglets from Sichuan Province. Further research is required to determine the exact role of PKoV variants in swine disease.

Many emerging viruses are of zoonotic origin and cause epidemics in humans after overcoming the interspecies barrier through mutation or recombination events. Intertypic recombination in H023/2009/JP implies the possibility of cross-species transmission of kobuviruses [23,28]. PKoV was not detected among 454 samples that were obtained from children with diarrhea in China [30]. Given that the frequency of recombination events in RNA viruses is relatively high, the possibility of zoonotic transmission among kobuvirus cannot be excluded.

PKoV is prevalent in both healthy and diseased pigs, and we suspect that at least two different PKoV types exist in piggeries. One type likely leads to gastroenteritis in pigs and possibly acts in combination with other enteric viruses; the other type probably causes subclinical infections in animals. Similar speculation has been mentioned by Verma *et* al. They also speculated that PKoV pathogenicity could be related to virus load and the presence of other enteric viruses, or kobuvirus might just be an endogenous passenger virus [25]. The difficulties in propagating PKoV *in vitro* limit our understanding of the growth kinetics and pathogenicity of this virus. Detection of PKoV in serum samples has been reported in Hungary and Brazil [19,27]. Further extensive epidemiological investigation of different PKoV strains from various regions would facilitate the understanding of its clinical and epidemiological characteristics.

## Conclusions

In conclusion, PKoV is endemic to Sichuan Province regardless of clinical conditions. High prevalence of this virus in piglets under the age of 4 weeks, and in pigs with diarrhea was observed. Genetic diversity of the virus within the same host and between different hosts was seen. For the first time, we provide evidence of PKoV recombination in the VP1 region, along with the existence of multiple PKoV strains in a single pig. These findings should contribute to understanding features of PKoV epidemics and genetic characteristics of the virus. Greater attention should be paid to this potential pathogen, and PKoV should be routinely tested for in diarrhea samples sent to diagnostic laboratories.

## Methods

### Specimens

A total of 163 porcine intestinal and fecal samples were collected during 2011–2012, covering 18/21 cities of Sichuan Province, China. The number of non-diarrheal and diarrheal stool was 51 and 112, respectively. In addition, 4 fecal specimens were obtained from apparently healthy Tibetan pigs in Kangding County, Ganzi Tibetan Autonomous Prefecture. The collections consisted of 93 samples from suckling pigs (< 4 weeks), 23 samples from weaned pigs (< 7 weeks), 17 samples from growing/finisher pigs (< 6 months), 30 samples from sows (> 1 year). One sample was obtained per pig and placed into a sterile specimen container. Of note, sampling procedures were approved by the National Institute of Animal Health Animal Care and Use Committee of Sichuan Agricultural University Ethics Committee (approval number 2010–020).

### RNA extraction and reverse transcription-PCR (RT-PCR)

Intestinal contents and fecal samples were prepared as 10% (wt/vol) intestinal/fecal suspensions with phosphate-buffered saline (PBS, pH 7.2-7.4) through vortex. The prepared sample suspensions were clarified by centrifugation (4000 r.p.m, 10 min, 4°C). Total RNA was extracted from a 300 μl starting volume of the centrifuged sample suspensions using TRIZOL reagent (TaKaRa Bio Dalian, CO., LTD.) according to the manufacturer’s instructions. The extracted RNA was resuspended in 30 μl RNAase-free water. Reverse transcription was carried out using a cDNA synthesis kit (TaKaRa Bio Dalian, CO., LTD.), and the cDNA was immediately used for amplification or stored at -40°C.

PKoV screening was assessed by amplifying a 495-bp fragment using previously reported primers specific for the 3D region of PKoV [20]: forward:5′-TGGACGACCAGCTCTTCCTTAAACAC-3′, reverse:5′-AGTGCAAGTGCAAGTCTGGGTTGCAGCCAACA-3′; Amplicons were visualized on a 1.5% ethidium-bromide-stained agarose gel under ultraviolet transillumination. Negative controls were analyzed in parallel with each primer set to discard contaminations and false positive.

### Polyprotein gene amplification and sequencing

Representative PKoV positive strains detected in this study were randomly selected for analyzing their VP1 sequences. A nested PCR was performed to amplify the VP1 region. Amplification of the PKoV VP1 partial sequence was conducted by using forward primer 5′-GTGGTATCCAAGCTCCTGGATTTC-3′ and reverse primer 5′-TGGCACGTCAGTAACCAGGCATT-3′ in the first-round PCR and forward primer 5′-GTCTCCAGCATTGAGTCTGG-3′ and reverse primer 5′- AGGGCGGACCACAGCAGCAACA-3′ in the nested PCR. The first-round PCR was aimed to amplify the PKoV polyprotein sequence, covered the full VP1 region, a portion of VP3, and a part of the 2A region. The nested PCR was designed to amplify the partial VP1 sequence, the amplicon size is about 706 bp.

The PCR was carried out by using PrimeSTAR Premix (TaKaRa Bio Dalian, CO., LTD.) containing high fidelity Taq DNA polymerase. After nested PCR, the reaction mixture was added with Taq polymerase (TaKaRa Bio Dalian, CO., LTD.) and incubated at 72°C for 30 min. In this way, deoxyadenosines could be added to 3′ end of the PCR products. PCR products were purified and cloned into the pMD-T-19 simple vector (TaKaRa Bio Dalian, CO., LTD.) prior to sequencing. Three clones were sequenced per sample.

First-round PCR products were directly subjected to sequencing in Sangon Biotech with primers used in the first-round PCR when strong target bands could been seen in the agarose gel electrophoresis of first-round PCR products. Interestingly, in two diarrheic samples the sequencing chromatograms showed that at specific positions alternative nucleotides could be found, suggesting that the two pigs were co-infected with more than one PKoV strains at the time of sampling. These first-round PCR products were cloned into the pMD-T-19 simple vector and the recombinant plasmid was transferred into *E.coil* DH5a competent cells. At least ten positive clones were sent for sequencing for each sample.

### Statistical analysis

The determined prevalence of PKoV in our study was analyzed statistically using Pearson’s chi-square test in SPSS 21.0 to find out whether it was correlated with stool conditions and age of pig. Moreover, *p* <0.05 was considered statistically significant, while a *p* value <0.001 indicated extremely marked statistical significance.

### Sequence and phylogenetic analysis

The obtained nucleotide sequences and deduced amino acid sequences (excluding primer pair sequences) were compared with other known kobuviruses in the GenBank. Sequence similarity analysis was performed with the aligned nucleotide and amino acid sequences by the Clustal W method using the Megalign 7.2 program of Lasergene software (DNASTAR, Madison, USA). The Phylogenetic tree was constructed based on nucleotide alignments using the MEGA 5.0 program [48]. Nucleotide sequences were aligned using the ClustalW method and the phylogenetic tree was carried out by the neighbour joining method and Kimura 2-parameter model assuming uniform rates of change among sites, reliability value at each node was assessed by bootstrap method with 1,000 replications.

### Recombination analysis

To detect possible recombination events among nucleotide alignment of the PKoV polyprotein sequences (1190 bp) from one pig, the Recombination Detection Program (RDP) was used to identify the possible parental sequences and recombinant strain [49]. The similarity between the putative parent strains and putative recombination could be showed in Simplot page using SIMPLOT version 3.5.1 [39]. Bootscan analysis was used to further investigate the potential recombination sites. We perfomed the software in the 2-parameter (Kimura) distance model and a sliding window of 200 nucleotides, step size of 20 bp.

## Competing interests

The authors declare that they have no competing interests.

## Authors’ contributions

Conception and design of the experiments: LC, LZ, WZG, ZWX; Samples colletion: LC, YCZ, ZWX, WYY. Experimental work: LC; LZ; YCZ; WYY; Sequence and data analysis: LC; LZ; YCZ; manuscript preparation: LC. All authors have read and approved the final manuscript.
